# Lipid polymorphism in chloroplast thylakoid membranes – as revealed by ^31^P-NMR and time-resolved merocyanine fluorescence spectroscopy

**DOI:** 10.1038/s41598-017-13574-y

**Published:** 2017-10-17

**Authors:** Győző Garab, Bettina Ughy, Pieter de Waard, Parveen Akhtar, Uroš Javornik, Christos Kotakis, Primož Šket, Václav Karlický, Zuzana Materová, Vladimír Špunda, Janez Plavec, Herbert van Amerongen, László Vígh, Henk Van As, Petar H. Lambrev

**Affiliations:** 1 Institute of Plant Biology, Biological Research Centre, Hungarian Academy of Sciences, Temesvári körút 62, H-6726 Szeged, Hungary; 2 Department of Physics, Faculty of Science, University of Ostrava, Chittussiho 10, CZ-710 00 Ostrava, Czech Republic; 3 MAGNEFY, Magnetic Resonance Research Facility (Wageningen NMR Centre), Wageningen University & Research, Stippeneng 4, 6708 WE Wageningen, The Netherlands; 4 Laboratory of BioNano Technology, Wageningen University & Research, Bornse Weilanden 9, 6708 WG Wageningen, The Netherlands; 5 Slovenian NMR Center, National Institute of Chemistry, Hajdrihova 19, Ljubljana, Slovenia; 6 EN-FIST Center of Excellence, Trg OF 13, Ljubljana, Slovenia; 7 Faculty of Chemistry and Chemical Technology, Večna pot 113, Ljubljana, Slovenia; 8 Laboratory of Biophysics, Wageningen University & Research, Stippeneng 4, 6708 WE Wageningen, The Netherlands; 9 MicroSpectroscopy Centre, Wageningen University & Research, Stippeneng 4, 6708 WE Wageningen, The Netherlands; 10 Institute of Biochemistry, Biological Research Centre, Hungarian Academy of Sciences, Temesvári körút 62, H-6726 Szeged, Hungary

## Abstract

Chloroplast thylakoid membranes contain virtually all components of the energy-converting photosynthetic machinery. Their energized state, driving ATP synthesis, is enabled by the bilayer organization of the membrane. However, their most abundant lipid species is a non-bilayer-forming lipid, monogalactosyl-diacylglycerol; the role of lipid polymorphism in these membranes is poorly understood. Earlier ^31^P-NMR experiments revealed the coexistence of a bilayer and a non-bilayer, isotropic lipid phase in spinach thylakoids. Packing of lipid molecules, tested by fluorescence spectroscopy of the lipophilic dye, merocyanine-540 (MC540), also displayed heterogeneity. Now, our ^31^P-NMR experiments on spinach thylakoids uncover the presence of a bilayer and three non-bilayer lipid phases; time-resolved fluorescence spectroscopy of MC540 also reveals the presence of multiple lipidic environments. It is also shown by ^31^P-NMR that: (i) some lipid phases are sensitive to the osmolarity and ionic strength of the medium, (ii) a lipid phase can be modulated by catalytic hydrogenation of fatty acids and (iii) a marked increase of one of the non-bilayer phases upon lowering the pH of the medium is observed. These data provide additional experimental evidence for the polymorphism of lipid phases in thylakoids and suggest that non-bilayer phases play an active role in the structural dynamics of thylakoid membranes.

## Introduction

In oxygenic photosynthetic organisms the light reactions of photosynthesis occur in the thylakoid membranes, flattened lipid vesicles, which separate the inner (lumenal) and outer (stroma side) aqueous phases. These membranes are densely packed with pigment-protein complexes and other constituents of the photosynthetic machinery. They contain the two photosystems (PSs), PSII and PSI, along with their associated light-harvesting antenna complexes (in green plants, LHCII and LHCI). They also embed the cytochrome b_6_f complex and some further constituents of the electron transport system, and the ATP-synthase. The operation of the electron transport system, linked to proton transport, is driven by the primary photochemical reactions and leads to the evolution of molecular oxygen, released to the atmosphere, and the synthesis of NADPH, carrying the reducing equivalents for CO_2_ fixation. The primary charge separation in the reaction centres and the consecutive vectorial charge transport also generate an energized state of the thylakoid membrane, an electrochemical potential gradient (proton-motive force, Δµ_H__+_), consisting of a transmembrane ΔpH (of 2–3 pH units) and an electric potential gradient (ΔΨ, of ~10^5^ V cm^−1^) - which are utilized for the synthesis of the energy-carrier molecule ATP^1^. The build-up of Δµ_H__+_ and its utilization via the ATP-synthase is warranted by the organization of the thylakoid membrane as a bilayer.

The bilayer organization of the membranes, viz., the impermeability of membranes to water and most water-soluble molecules and to ions, is the basic property of all energy-converting biological membranes^2^. In the light of this strong restriction on the functional state of the energy-converting membranes, it is not easy to understand that the major lipid species in thylakoid membranes, constituting about half (45–55%) of the total lipid content, is the non-bilayer lipid monogalactosyl-diacylglycerol (MGDG)^3^. Non-bilayer or non-lamella forming lipids, for their conical shapes^4^, are not capable to self-assemble into bilayers in aqueous media under physiologically relevant conditions^2,5^; instead, they are assembled into different non-lamellar or non-bilayer lipid phases, such as the inverted hexagonal (H_II_), isotropic and cubic phases. Only about the other half of the thylakoid membrane lipids, digalactosyl-diacylglycerol (DGDG, ~25–30%), sulfoquinovosyl-diacylglycerol (SQDG, ~10–15%) and phosphatidylglycerol (PG, ~10–15%), exhibiting cylindrical shapes, are bilayer lipids. While the significance of specific lipid-protein interactions, e.g. between MGDG and the key protein complexes of the thylakoid membrane^6–9^ should not be ignored, explanation must be offered for the behaviour of bulk lipids in the bilayer: about 60% of the total thylakoid lipids are found in a fluid-like phase at room temperature^10^.

The dominance of lipids with non-bilayer phase propensity is not unique to thylakoid membranes but holds true for other energy-converting membranes, with basically different lipid and protein compositions in mitochondria and retina - containing no galactolipids and no pigment-protein complexes. Albeit at lower concentrations, probably all biological membranes contain non-bilayer lipid species^4^. However, their role in the bilayer biological membranes remains enigmatic.

When testing the general features of biological membranes, one could easily arrive to the conclusion that none of the basic features of biological membranes appears to require the presence of non-bilayer lipids, and virtually all basic functions – the insulating properties, the lateral diffusion of mobile components, the embedding of integral proteins in a two-dimensional matrix - can be and have been thoroughly studied in artificial membranes containing only bilayer lipids^11^. There are, however, specific features in which bilayer lipids appear to offer no solution. For instance, lipid bilayers tend to repel each other, preventing their fusion^12^. Although the role of specific proteins (such as the SNARE proteins) in the fusion of membrane vesicles is well established, non-bilayer lipids might play a role in forming intermediate structures that are involved in membrane junctions and fusion, and protein transport across membranes^13–15^. Non-bilayer lipids are capable of forming fusion channels and are required for the functioning of SNARE^16^. In chloroplast and cyanobacterial thylakoid membranes IM30/Vipp1 protein plays a key role in membrane fusion; as suggested by *in vitro* experiments, and local formation of a H_II_ lipid phase is thought to be the first event during the interaction of this protein with the membrane^17^.

Concerning the thylakoid lipids, numerous data have shown that *in vitro* and in the absence of mature pigment-protein complexes they are capable of forming different non-bilayer structures^2,18–20^. Recently, it has been demonstrated by molecular dynamic model calculations that a bilayer constituted of a thylakoid lipid mixture is instable and tends to form non-bilayer structures; further, stalks can be formed between stacked bilayers^5^. However, it is commonly agreed that self-assembled lipid:LHCII membranes and functional thylakoid membranes are composed of just one phase, the bilayer^2,18,21,22^, similar to the general membrane models.

Several membrane models have been proposed in the past years to answer one of the basic questions of membrane biology, viz., concerning the roles of non-bilayer lipids in the bilayer membranes. The models agree on two main points: (i) While non-bilayer lipids in different organisms appear to participate in essential lipid-protein interactions, the general answer to their roles in the bulk lipid bilayers of biological membranes must be related to their non-lamella-forming property, rather than their chemical composition. (ii) The biological membranes are organized in bilayers, and non-bilayer phases are present only locally and transiently in the bilayer membranes. As stressed in the review of van den Brink-van der Laan *et al*.^23^: “All membranes contain these lipids in large amounts. Yet, the lipids in biological membranes are organized in a bilayer”.

In the membrane model proposed by de Kruijff^24^, it is suggested that non-bilayer lipids increase the lateral pressure in the acyl chain region of the bilayer, and decrease the lateral pressure among the lipid headgroups, affecting protein functions. In an extension of this model (which will be referred to as lateral pressure model or LPM), van den Brink-van der Laan *et al*.^23^ suggest that “non-bilayer lipids stimulate membrane binding of peripheral membrane proteins and affect the stability of (oligomeric) complexes of integral membrane proteins via changes in the lateral pressure”, an effect shown e.g. on a potassium channel protein. The same authors also point out that since non-bilayer lipids prefer organization in curved structures, the bilayers containing lipids with high non-bilayer propensity will assume a frustrated state. This suggests that non-bilayer lipids play a prominent role in the structural dynamics of membranes in the lateral direction.

The flexible surface model (FSM), proposed by Brown^25^, also “challenges the standard model (the fluid mosaic model [of Singer and Nicolson^26^, Nicolson^27^]) found in biochemistry texts”. The FSM “describes the balance of curvature and hydrophobic forces in lipid–protein interactions” in the bilayer, an effect demonstrated on rhodopsin. A key prediction of the FSM is that “the non-lamellar-forming tendency of the membrane lipids modulates the protein energetics”. This can be very strong once the lipid composition is such that it is close to a lamellar–hexagonal phase boundary, i.e. the lipid mixture has strong non-bilayer propensity and thus displaying stressed features. This, similarly to LPM, can be interpreted in terms of increased structural flexibility in the lateral direction.

The model proposed by Garab *et al*.^28^ is based on experimental data showing that LHCII is capable of destroying the H_II_ phase of purified MGDG and forcing these lipids into a bilayer structure^18^. The two compounds - LHCII, the most abundant membrane protein in the Biosphere^29^, and MGDG, the most abundant polar lipid on Earth^30^ - have been shown to assemble in large bundles of stacked membranes^31^. In these macroassemblies, LHCII self-aggregates to packing density comparable to that in the thylakoid membranes, preventing the formation of non-bilayer structures inside the membrane^18^. These observations led to the hypothesis that – upon loosening the local packing density of proteins (e.g. during heat stress^32^), and thus the transient formation of non-bilayer structures – the lipids readily segregate, while upon the “arrival” of more membrane proteins these lipids can (re-)enter the membrane^28^. This model assumes a close association of the bilayer and non-bilayer lipid phases and a dynamic exchange is hypothesized between them, which will thus be referred to as DEM (dynamic exchange model). This model assumes the co-existence of the bilayer phase and (a) non-bilayer lipid phase(s), other than e.g. in the form of lipid droplets, which are physically separated from the membranes, often seen in electron microscopic images^33,34^. Via dynamic exchange, non-bilayer lipids may lend additional structural flexibility to the membranes, in the transmembrane direction.

The DEM gained support from ^31^P-NMR experiments on isolated, fully functional, intact thylakoid membranes, which exhibited, in addition to the lamellar phase at around −10 ppm, intense resonances between about 5 and 0 ppm^35^. One of these latter resonances, peaking at around 4 ppm, was assigned to an isotropic signal given rise by PG molecules found in non-bilayer structures. It was also observed that the signature of the lamellar phase vanished already at around 20 °C, despite the fact that the bilayer functions were retained. This loss was attributed to communication between the bilayer and the non-bilayer lipid phases. In other terms, the non-bilayer structures remained in contact with the membrane via temperature-dependent exchange of lipids between the two phases. The conclusion on the heterogeneity of lipid phases has been substantiated by applying fluorescence spectroscopy and lifetime measurements using MC540^36^. MC540, being sensitive to variations in the local dielectric constant of the lipidic phase, has been shown to be capable of detecting changes in the lipid packing and phases^37^. Strong heterogeneity was reflected by fluorescence lifetime data, which could be assigned to either a broad distribution of lipid-packing states or to three discrete phases, with two fractions found clearly in lipidic microenvironments^36^. These ^31^P-NMR and MC540 data have thus shown that plant thylakoids contain non-bilayer structures and thus these membranes cannot be portrayed as composed of a single, bilayer phase. However, our understanding of the lipid polymorphism of thylakoid membranes has remained rudimentary.

In this work, by employing time-resolved MC540 fluorescence spectroscopy, we show the presence of multiple distinct lipidic microenvironments, and our ^31^P-NMR measurements reveal the existence of three non-bilayer phases in addition to the bilayer phase and demonstrate substantial reorganizations when the membranes are exposed to different physico-chemical environments.

## Results and Discussion

### Time-resolved MC540 fluorescence spectroscopy signature of thylakoid membranes

**Figure 1:**
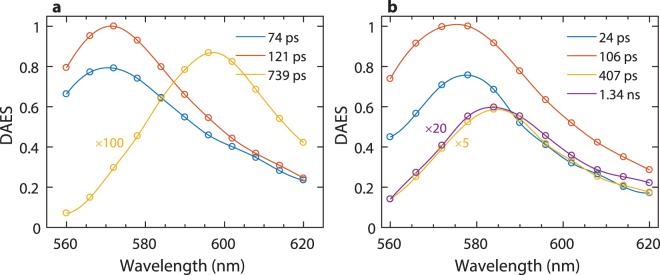
Decay-associated fluorescence emission spectra (DAES) of MC540. (**a**) MC540 (10 μM) in resuspension buffer; (**b**) MC540 (10 μM) in thylakoid membranes at lipid:dye ratio approximately 50:1. The DAES were obtained by global lifetime analysis of the fluorescence decay traces recorded with 540 nm excitation. Errors in the lifetimes were <7%. For easier comparison, some spectra are scaled, as indicated.

The fluorescence of MC540 in the resuspension buffer decayed with two lifetimes, 70 ps (45% amplitude) and 120 ps (55%), very close to the 110 ps lifetime observed in water^38^. The decay-associated emission spectra (DAES) of both components showed a maximum at 572 nm (Fig. 1a). An additional 800 ps component with an amplitude of less than 1%, peaking at ~600 nm, likely originates from “nonfluorescent” MC540 dimers^39^. Since the lifetimes and spectra of the two major components were similar to each other, they could be combined into a single lifetime of 104 ps, albeit the fit was inferior (Supplementary Fig. 1). When MC540 was added to thylakoid membranes the fluorescence yield increased substantially and four lifetimes, in the range of 24 ps to 1.3 ns, were necessary to describe the decay (Fig. 1b, see also Supplementary Fig. 2). The stationary fluorescence spectrum shifted from 572 nm to 580 nm (Supplementary Fig. 3). The DAES peak positions also exhibited marked heterogeneity: the 24 ps component (with ~40% amplitude) peaked at 578 nm, the largest-amplitude 106 ps component (~53%) – at 575 nm, and the longer-lifetime components (407 ps – ~6% and 1.34 ns – ~1%) – at 584 nm. Earlier, the shortest (113–214 ps) lifetime component of MC540 in the thylakoid membrane was assigned to originate from molecules that were close to or remained in the aqueous phase^36^. While in the case of the 120 ps DAES component, exhibiting a marked broadening on the short-wavelength side, some contribution from free MC540 cannot be ruled out, for the 24 ps component the sizeable bathochromic shift compared to the aqueous-phase DAES peak rules out this possibility (Fig. 1). (Significant contributions from free MC540 molecules can also be ruled out based on absorbance spectroscopy data (Supplementary Fig. 4)). It can thus be concluded that in MC540-stained thylakoids a large fraction of the dye molecules are found in lipid phases or lipid-containing domains close to the aqueous phase, rather than in the aqueous phases as proposed previously^36^. Further, the existence of the two slow DAES components, which can be ascribed to MC540 associated more closely with lipids and less exposed to water, also indicate a heterogeneity in the lipid phases. Although the nature of these lipid phases, or lipid-containing domains, remains to be clarified, it can be concluded that our MC540 data reveal higher heterogeneity in the thylakoid membranes than previously thought^36^.

### ^31^P-NMR signatures of intact thylakoid membranes and the stability of lipid phases

**Figure 2:**
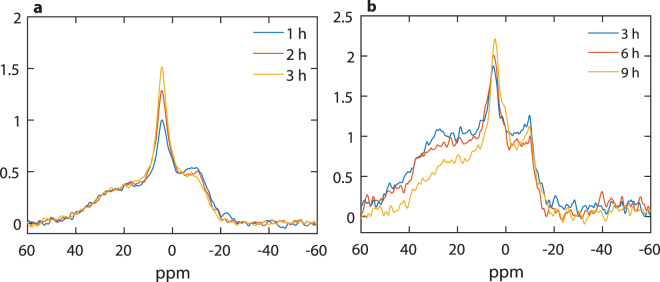
^31^P-NMR spectra of isolated spinach thylakoid membranes. Thylakoids isolated and suspended in a sorbitol-based isotonic medium (6.3 mg/ml chlorophyll (a + b) content (Chl)), the first 3 hours are plotted (**a**); thylakoids isolated and suspended in a NaCl-based isotonic medium (4.0 mg/ml Chl) (**b**). The spectra were recorded for 1 hour, using, the 300 MHz NMR spectrometer. In (**b**), the measured spectra are averaged for 3-hour periods partly because of the lower signal-to-noise ratio in the NaCl-based sample, which is due to dielectric and conductive losses in the rf efficiency, and partly because of the larger stability of the sample compared to the sorbitol-based sample. For further details, see Materials and methods.

The ^31^P-NMR spectrum of freshly isolated spinach thylakoid membranes, recorded at 5 °C, agrees reasonably well with the earlier reported spectra measured under similar conditions^35^. It can be seen that signals originate from several different chemical environments of the phosphorous nucleus (Fig. 2a). The signals in these samples have been shown to originate predominantly from PG of the thylakoid membranes. As was observed earlier for intact and TRIS-washed thylakoid membranes^35,40^, a clearly discernible peak is present at −10 ppm, originating from the lamellar phase. Also, as in the earlier measurements, the spectrum is dominated by the intense resonances around 4 ppm, here peaking at 4.8 ppm – ascribed to an isotropic, non-bilayer phase; the resonances between 20 and 40 ppm were proposed to belong to the lamellar phase, representing its shoulder at low field, ~30 ppm^35^.

It is interesting to observe that the peak at 4.8 ppm increases during consecutive measurements, doubling its intensity in about 5 hours (not shown). At the same time, the signals in all other regions are somewhat diminished. These changes, the magnitude and rate of which depend on the isolation conditions and which vary from batch to batch, might originate from subtle membrane reorganizations affecting e.g. the mobility of lipid molecules in this non-bilayer structure. Isolated thylakoid membranes, in our hands, under similar conditions (stored in dark at high concentration and around 5 °C), retain their structural and functional parameters for at least 4–8 hours. Structural parameters have been tested e.g. by circular dichroism and small-angle neutron scattering (SANS), carrying information about the chiral macro-organization of protein complexes^41^ and the periodic lamellar organization of thylakoid membranes, respectively^42^. Routinely monitored functional parameters, such as the chlorophyll-a fluorescence and P700 absorbance transients – testing the activity of PSII and PSI, respectively - are also reasonably robust; and no decline is noticed in the capacity of membranes to build up and maintain the transmembrane electric potential gradient^43^, a parameter that depends on the impermeability of the bilayer. Here, we tested the variations of circular dichroism and 77 K fluorescence emission spectra (Supplementary Figs 5 and 6, respectively) and of chlorophyll-a fluorescence transients (Supplementary Table 1) during 1, 3, 6 and 8 hrs of storage of the membranes compared to the freshly prepared samples, and found no clue for the gross variations in the lipid phase behaviour. This instability may reflect the inherent dynamic features of the lipid phases and thus warrants further investigations.

Figure 2b shows that changing the microenvironment of membranes - using a NaCl-based isotonic isolation- and suspending medium, instead of the widely used sorbitol-based medium - significantly affects both the ^31^P-NMR signature and the stability of the lipid phases. While the dominance of the isotropic peak was retained, the relative amplitude of the −10 ppm lamellar peak increased and the intensity of the resonances at around 30 ppm rose to the level of the lamellar phase signal - ruling out its origin as the low-field shoulder of the bilayer phase at −10 ppm. As also suggested by data below, this signal can be tentatively ascribed to the H_II_ phase – which might be found, and in fact is often seen by electron microscopy, in the stromal liquid^33,34^.

It can also be seen that the temporal stability of the membranes in the NaCl-based medium is significantly improved compared to that in the sorbitol-based medium; this is particularly well discerned in the region of the isotropic peak at 4.8 ppm, which increased only by about 20% in 9 hours. This peak also contained a shoulder on the low-field side, which might be responsible for the apparent shift of the peak to 4.2 ppm after 9 hours. Recently, SANS experiments have shown that the *in vivo* ultrastructure of thylakoid membranes is much better preserved in NaCl-based media than in sorbitol, which loosens up the ultrastructure of thylakoid membranes; and their structural flexibility is more similar to that in leaves^44^. It can thus be inferred that the lipid phases in thylakoids in their native environments are more robust than what is observed in the sorbitol-based medium (Fig. 2a) and what is reflected by their strong temperature dependence^35^. Nevertheless, it is very likely that thylakoid lipid phases *in vivo* possess significant dynamic features, an inherent structural flexibility similar to that observed in the presence of 400 mM NaCl.

The dominance of the isotropic lipid phase suggests an origin associated with a structural element of the thylakoid membrane system. In fusion channels the lipids are in an isotropic phase^13^. For the 4.8 ppm peak in the thylakoid membranes, a similar origin can be proposed. In chloroplast thylakoid membranes of vascular plants the granum and stroma thylakoid membranes appear to be fused in the junction regions^45–47^.

### Effects of 2 M sucrose and the catalytic hydrogenation of thylakoid lipids

It has been found that co-solutes at high concentrations extrude significant amounts of lipid molecules from the membrane, forming the H_II_ phase, and, consequently, the photosynthetic functions, in particular that of PSII, gain improved thermal stability^48,49^. These observations clearly demonstrate the crucial role of lipid molecules and their segregation capability in determining the stability and structural dynamics of thylakoid membranes.

**Figure 3:**
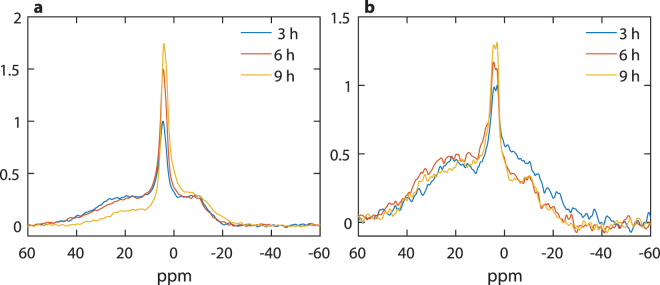
^31^P-NMR spectra of isolated spinach thylakoid membranes. Effects of 2 M sucrose (**a**) and the homogenous catalytic hydrogenation of the thylakoid membranes (**b**), with 5.7 mg/ml and 3.8 mg/ml Chl contents, respectively. The spectra were recorded for 1 hour, using, the 300 MHz NMR spectrometer, and are averaged for 3-hour periods. For further details, see Materials and methods.

When thylakoid membranes are suspended in a reaction medium containing 2 M sucrose, a co-solute inducing a phase separation of lipids and raising the stability of PSII^49^, the intensity of the low-field resonances at around 30 ppm rises relative to the lamellar phase (Fig. 3a). This and the stability of the lamellar phase are similar to that in the presence of 400 mM NaCl. However, the isotropic peak, at 4.7 ppm, appears to be less stable – in time its amplitude increases and its position shifts to higher field by more than a half ppm.

Homogenous catalytic hydrogenation of the membrane lipids, i.e. a reduction of the level of total lipid unsaturation, in this case, by 23.6% (see Supplementary Table 2) exerts distinct effects on the ^31^P-NMR signature and the stability of the lipid phases of thylakoids (Fig. 3b). Most remarkably, the isotropic peak splits into two, with positions at about 4.2 and 3.1 ppm. These signals dominate the spectrum. The emergence of the 3.1 ppm peak upon saturation of double bonds of fatty acyl constituents of the membrane lipids corroborates our conclusion that the resonances in this region in our samples originate from PG. Its variation may reflect changes in its microenvironment and the mobility of the molecules. It is also interesting to note that rigidifying the membranes appears to stabilize the non-bilayer phase giving rise to the peak at around 25–30 ppm, rather than stabilizing the lamellar phase.

**Figure 4:**
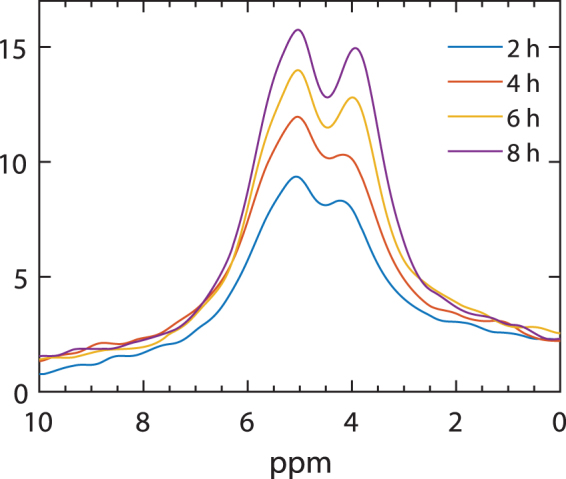
^31^P-NMR spectra of spinach thylakoid membranes treated with 2 M sucrose. The spectra were recorded in a 600 MHz spectrometer (Bruker) in two hours intervals, following the co-solute treatment; Chl content, 3.1 mg/ml.

Splitting of the isotropic peak, in the 600 MHz spectrometer, can also be observed in the absence of hydrogenation, in co-solute-treated thylakoid membranes (Fig. 4). This also explains the apparent shift of the 4.7 ppm peak, which can be accounted for by the gradually increased contribution from a peak at around 3.9 ppm, a signal present already at the beginning of the measurements. Earlier, a peak at 2.6 ppm was ascribed to inorganic phosphate liberated from the lumen^35^ but this seems highly unlikely given the fact that 2 M sucrose did not impair the photosynthetic functions. Hence, the peak at 3.9 ppm should rather be assigned to a lipid phase, probably another isotropic non-bilayer phase arising from a different microenvironment. We propose that this signal originates from lipids extruded from the membranes. The extruded lipids might be bound to lipocalin or lipocalin-like molecules, where they may form a non-bilayer structure, as proposed by Garab *et al*.^50^. The thylakoid lumen contains the chloroplastic lipocalin, CHL^51,52^, as well as the lipocalin-like enzyme, VDE, the violaxanthin deepoxidase^53,54^. A lipocalin on the stromal side, the zeaxanthin epoxidase (ZE)^53,54^, might play a similar role. CHL has been shown to accumulate in response to temperature stress and to protect thylakoid lipids against reactive oxygen species^55^, and has been shown to be involved in a mechanism for sustained photoprotective non-photochemical quenching (NPQ) of the first singlet excited state of chlorophyll-a^56^. The temperature-induced lipocalin (TIL) has been proposed to be involved in the defence mechanisms against heat, cold, oxidative and salinity stresses^57^. VDE and ZE are key enzymes of the xanthophyll cycle (XC), a defence mechanism against high light^54,58^. Note that, albeit at different rates, both peaks gain intensity with the progress of time; further, at this resolution and S/N, we see no indication of a peak at 0 ppm, which was reported earlier^35^

### Low-pH induced lipid-phase reorganizations revealed by ^31^P-NMR

Acidification of the lumen has been shown to play a key role in the photoprotective regulatory mechanisms of photosynthesis^59–61^. The energy-dependent NPQ depends on the transmembrane ∆pH both via the PsbS protein and the XC. Low-pH and transmembrane-∆pH induced structural changes have been thoroughly documented in the literature^62–69^. Recently it has been shown using SANS that low pH induces significant variations in the periodic organization of chloroplast thylakoid membranes^69^. The observed reorganizations, shrinking of the lamellar repeat distances and the diminishment of the periodic order of the thylakoid membranes closely resembled the light-induced, ∆pH-dependent reorganizations in isolated thylakoid membranes^70^. To our knowledge, the role of lipids in low-pH induced thylakoid reorganizations has not been investigated.

**Figure 5:**
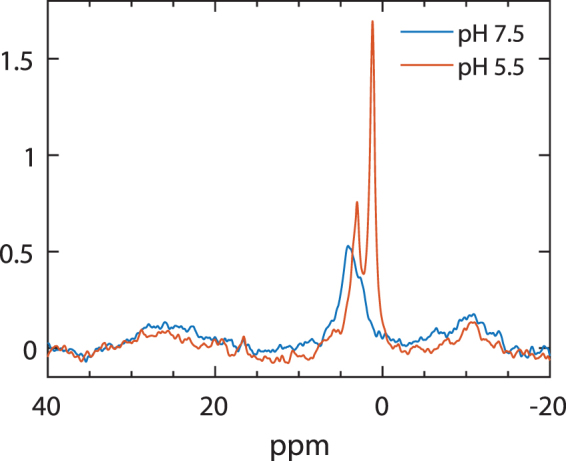
The effect of low-pH on the ^31^P-NMR spectrum of isolated thylakoid membranes. Freshly isolated thylakoid membranes, suspended in a sorbitol-based medium at pH 7.5 (9.8 mg/ml Chl) and 5.5 (13.1 mg/ml Chl) recorded on the Agilent 600 MHz NMR spectrometer at 5 °C.

Figure 5 shows that low pH induces significant changes also in the ^31^P-NMR of thylakoid membranes. The spectra show that the changes do not or only marginally affect the lamellar phase, at −10 ppm, and the non-bilayer phase at around 30 ppm. As noted above, these latter resonances, which are better resolved in the 600 MHz spectrometer than in the 300 MHz setup, can tentatively be assigned to a H_II_ phase, in reasonable accordance with their observed asymmetry and by analogy to a model system producing a peak of H_II_ origin at 20 ppm^71^. The identification of the lipid phase associated with this signal is beyond the scope of the present study.

The prominent low-pH-induced changes occur in the region of isotropic phases: the peak at 4.1 ppm shifts to 3.1 ppm, and the shoulder at 2.1 ppm shifts to 1.1 ppm and gains a very high intensity. Very similar changes are observed in thylakoid membranes in the NaCl-based medium, where the peaks at pH 7.5 and 5.5 are found at about 4.2 and 2.8 ppm, and 4.0 and 2.1 ppm, respectively (Supplementary Fig. 7). The shifts can be explained by pH effect on the chemical anisotropy^72^. The emergence of the peak on higher field side, however, evidently reflects significant reorganizations in the lipid phases. It is thus tempting to speculate that the generation of this intense non-bilayer phase by low pH plays a role in the operation of XC. VDE, the key XC enzyme is activated at low pH^58^ and its functioning depends on the presence of non-bilayer lipid phase^73^. VDE is responsible for the deepoxidation of violaxanthin of LHCII (located in the bilayer) to antheraxanthin and zeaxanthin^58,74^. The epoxidation of zeaxanthin to violaxanthin, is performed by the zeaxanthin epoxidase (ZE); this enzyme is found in the stromal-side aqueous phase. As discussed above, VDE is a lipocalin-like water-soluble protein and ZE is a lipocalin protein, and as such they are capable of interacting with lipid molecules^51^. VDE has been proposed to enter the membrane in the region containing a non-bilayer phase^54^. Alternatively, it may remain in the lumen, where it is proposed to bind lipid molecules and to form a non-bilayer phase that is associated with the bilayer^50^. In either case, the low-pH induced changes in the ^31^P-NMR spectrum of thylakoid membranes might indicate reorganizations affecting the phase behaviour of lipids. The appearance (or enhancement) of the isotropic phase at around 1.1 ppm (in sorbitol) and 2.1 ppm (in NaCl) is likely to be identical with the non-bilayer phase that is required to the functioning of VDE. By this means DEM might be Assisted by Lipocalins (DEMAL). With the use of sensitive spectrometers and lipocalin mutants it might in the future be possible to prove or disprove this membrane model.

## Summary and Concluding Remarks

**Figure 6:**
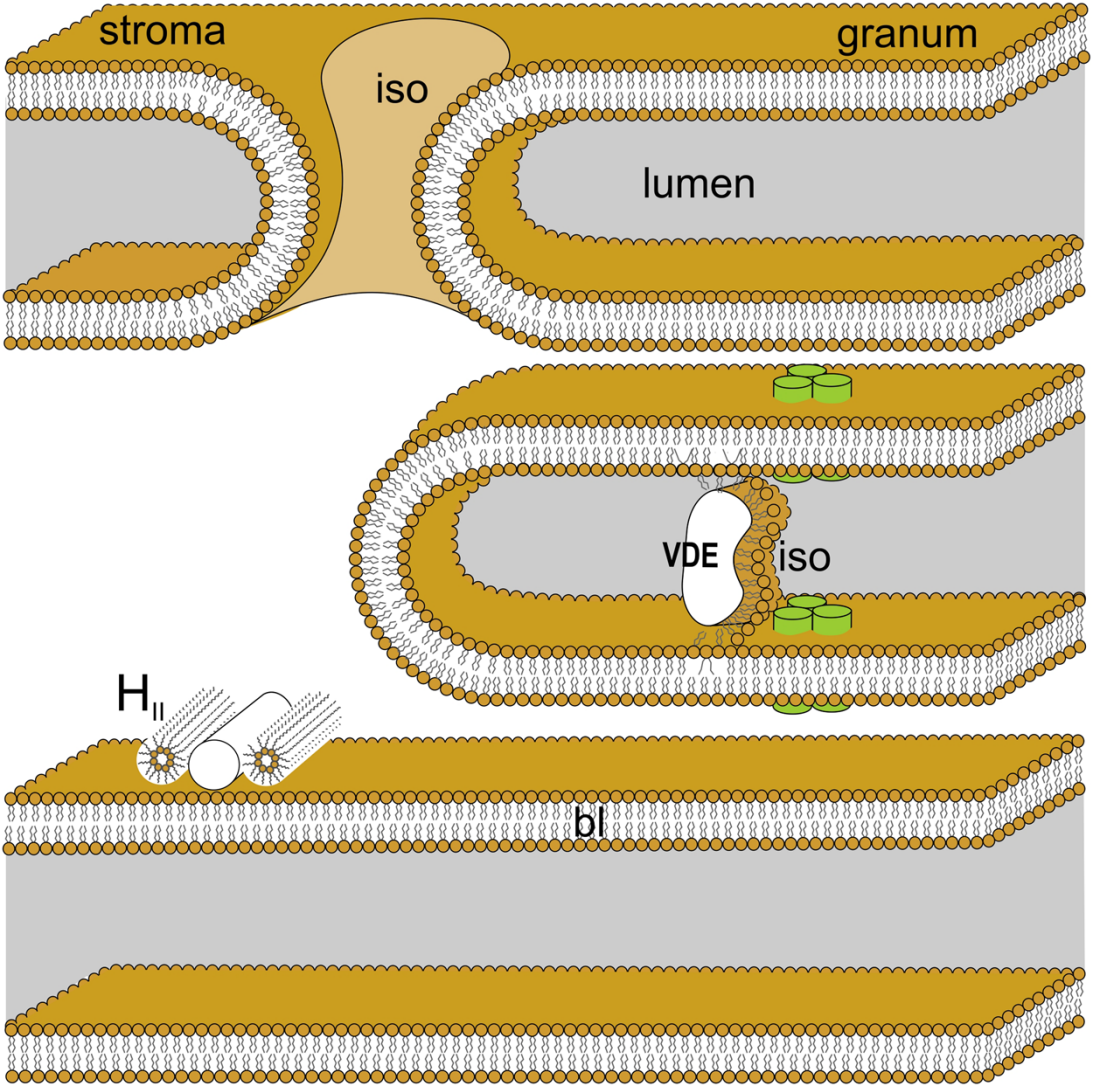
Schematic representation of thylakoid membranes of vascular plants – showing the tentative assignments of the lipid phases detected by ^31^P-NMR. Lipid phases: the basic bilayer (bl) structure, the non-bilayer, isotropic phases (iso) associated with the fusion of granum and stroma thylakoid membranes and with the lumenal lipocalin proteins,VDE and CHL, as well as the H_II_ phase in the stroma – possibly also associated with membrane-associated proteins and loosely attached to the membrane. The figure is not to scale; for simplicity, CURT1 proteins, which are enriched in the end-membranes of thylakoids and maintain the extreme curvature of membranes at the margins^75^, are omitted. Membrane-intrinsic proteins are symbolized by trimeric LHCIIs (green bars).

In this work, by using ^31^P-NMR and time-resolved MC540 fluorescence spectroscopy we provided additional experimental evidence on the polymorphic phase behaviour of the lipids in isolated spinach thylakoid membranes. With regard to the marked lifetime- and spectral heterogeneity of MC540, our data show the existence of different lipid domains, with different dielectric environments. Here only very hypothetical assignments can be given. Two components, with short lifetimes and small red shifts compared to the dye in the reaction medium, appear to originate from domains closer to (or partly mixed with) the aqueous phase – one of them might originate from H_II_ phase. The component with the longest lifetime and large red shift probably originates, with an evidently very low partition, from the bilayer; and the component with intermedier lifetime might be assigned to the isotropic phase. In ^31^P-NMR, in addition to the bilayer phase, we detected three different phases, which are tentatively ascribed to (i) H_II_ phase in the stromal-side aqueous phase; (ii) isotropic phase in the junction region of the granum and stroma thylakoid membranes; and (iii) another isotropic phase that is associated with lipocalins (VDE and CHL, on the lumenal side, as well as ZE on the stromal side) – a phase which might play significant roles in different regulatory mechanisms. Figure 6 summarizes schematically these hypothetic assignments.

In general, variations in the ^31^P-NMR signatures and in the temporal stability of the different lipid phases, upon exposing the membranes to different physico-chemical environments and by saturating double bonds of lipid fatty acyls, suggest that non-bilayer lipid phases contribute significantly to the structural dynamics of thylakoid membranes. Given the large abundance of non-bilayer lipids in all energy-converting membranes, similar to thylakoids, isolated plant thylakoid membranes - with their robust but still flexible ultrastructure, the availability of mutants affecting their lipid composition, lipocalin contents and membrane ultrastructure and with the wealth of information on their functions and key regulatory mechanisms - might serve as a model system toward the better understanding of molecular architecture and structural dynamics of biological membranes containing large amounts of non-bilayer lipids and possibly forming non-bilayer lipid phases.

## Materials and Methods

### Isolation of thylakoid membranes

Thylakoid membranes were isolated as described earlier^35^ with minor modifications. Dark-adapted leaves were homogenized in a medium containing 50 mM Tricine (pH 7.5), 5 mM MgCl_2_ and 5 mM KCl and 400 mM sorbitol or 400 mM NaCl for sorbitol- or NaCl-based preparations. The suspension was filtered through 4 layers of cheese cloth and centrifuged for 2 min at 400 g. Next, the supernatant was centrifuged for 10 min at 6,000 g. The chloroplasts were osmotically shocked in a hypotonic medium containing 50 mM Tricine (pH 7.5), 5 mM MgCl_2_ and 5 mM KCl for 10 s followed by the immediate addition of the same medium supplemented with double-strength osmoticum (800 mM sorbitol or 800 mM NaCl) before centrifugation for 10 min at 6,500 g. The pellet was finally resuspended in the original reaction medium. In experiments on the effect of sucrose as co-solute, after the osmotic shock, sorbitol was replaced with 2 M sucrose and the membranes were additionally washed and suspended in this medium. Chl content of the samples was determined according to Porra *et al*.^76^. All these procedures were performed under dim light and corresponding sample preparation on ice.

For the homogenous catalytic hydrogenation, thylakoid membranes were resuspended in 150 ml 50 mM HEPES buffer (pH 7.0) and supplemented with 0.8 M sorbitol. Hydrogenation, using Pd(QS)_2_ (Palladium Di (sodium alizarinmonosulfonate), was performed as described earlier^77,78^. After hydrogenation the sample was centrifuged for 10 min at 6,500 g and resuspended in the sorbitol-based reaction medium. Lipid analysis was performed as described by Vígh *et al*.^77^.

### Lipid isolation and fatty acid analysis

Lipids were extracted from 150 µl of isolated thylakoid membrane according to standard lipid-analytic methods^79^. The esterification of the fatty acids was done in 2.5% HCl containing methanol by incubating at 85 °C for 3 hours. Fatty acids were analysed on Supelco SP2330 capillary columns in a Hewlett Packard HP6890 gas chromatography equipment as described earlier^80^.

### MC540 labelling of the thylakoid membranes

For MC540 experiments 60 μg chlorophyll from sorbitol-based isolated thylakoid membranes was mixed with 10 μM MC540 and incubated for 30 min at room temperature. The mixture was diluted in 250 µl resuspension buffer (50 mM Tricine (pH 7.5), 5 mM MgCl_2_, 5 mM KCl, 400 mM sorbitol) and centrifuged for 15 min at 15,000 g. The pellet was resuspended in the same buffer before measurements. Incorporation of the dye in the thylakoid membranes was verified by absorption spectroscopy (Supplementary Fig. 4); it led to an approximately 30 nm bathochromic shift of the main absorption bands of MC540 (Supplementary Fig. 1b). These data also show that the excess amount of the dye was removed by washing the MC540-stained thylakoid membranes.

### Time-resolved fluorescence

Excited-state decay kinetics were measured at room temperature, using a FluoTime 200 spectrometer (PicoQuant, Germany) equipped with a microchannel plate detector (Hamamatsu, Japan) and a PicoHarp 300 TCSPC set-up (PicoQuant) previously described in detail^81^. In brief, a WhiteLase Micro supercontinuum laser (Fianium, UK) was used as a source of 540 nm excitation pulses. Fluorescence emission was detected through a monochromator in a wavelength range between 560 and 620 nm with 6 nm step size and binned in 4 ps time channels. Fluorescence decays were recorded at room temperature. Samples were diluted to OD 0.015 at the excitation wavelength in 1.5 mm path-length flow cell. The instrument response function (IRF) was measured at the excitation wavelength using 5% Ludox as scattering solution. The width of IRF was 40 ps. Global lifetime analysis of the fluorescence decays and iterative convolution with the measured IRF was performed using homebuilt MATLAB routines.

### ^31^P-NMR measurements

^31^P-NMR spectra were recorded at 5 °C on Avance-300 wide-bore (Bruker, Germany), Avance-600 (Bruker, Germany) and DD2 600 (Agilent, U.S.A.) spectrometers tuned at the resonance frequency of the ^31^P nucleus, with 20 mm and 5 mm outer diameter tubes containing about 15 ml and about 1.2 ml of concentrated thylakoid suspension for the 300 and 600 MHz spectrometers, respectively. The temperature was controlled within 0.1 °C; spectra were recorded using a 40° rf pulse, an interpulse time of 0.5 s and no^1^H-decoupling was applied. Stirring of the sample had no noticeable effect on the spectra, indicating that, evidently because of the very high density of the suspension, no significant magnetic orientation of membranes occurred.

In the experiments using the 600 MHz spectrometers each spectrum was recorded over approximately 2 hrs. Spectra were recorded unlocked. Chemical shifts were referenced externally to 85% H_3_PO_4_ in H_2_O (δ_P_ = 0 ppm). To estimate the effect of magnet drift six spectra were recorded on a single sample over 12 h using D_2_O in a coaxial insert for lock, which showed the drift to be negligible compared to the line width over the experiment time.

### Data Availability

The datasets generated during and/or analysed during the current study are available from the corresponding author on reasonable request.

## Electronic supplementary material

Supplementary Inforamtion
