# Supplementary material for: Lipid polymorphism in chloroplast thylakoid membranes – as revealed by 31P-NMR and time-resolved merocyanine fluorescence spectroscopy
Source: Sci Rep. 2017 Oct 17;7:13343. doi: 10.1038/s41598-017-13574-y (PMC5645462; doi:10.1038/s41598-017-13574-y)
Supplement: Supplementary file 1 — Supplementary Inforamtion [file 41598_2017_13574_MOESM1_ESM.pdf]

Supplementary Information

Lipid polymorphism in chloroplast thylakoid membranes – as revealed by ^{31}P-NMR and time-resolved merocyanine fluorescence spectroscopy

Győző Garab[1,2*], **Bettina Ughy**[1], **Pieter de Waard**[3], **Parveen Akhtar**[1], **Uroš Javornik**[4], **Christos Kotakis**[1], **Primož Šket**[4,5], **Václav Karlický**[2], **Zuzana Materová**[2], **Vladimír Špunda**[2], **Janez Plavec**[4,5,6], **Herbert van Amerongen**[7,8], **László Vígh**[9], **Henk Van As**[3,7*], **Petar H. Lambrev**[1*]

[1]Institute of Plant Biology, Biological Research Center, Hungarian Academy of Sciences, Temesvári körút 62, H-6726 Szeged, Hungary

[2]Department of Physics, Faculty of Science, University of Ostrava, Chittussiho 10, CZ-710 00 Ostrava – Slezská Ostrava, Czech Republic

[3]Magnetic Resonance Research Facility, Wageningen University & Research, Stippeneng 4, 6708 WE Wageningen, The Netherlands

[4]Slovenian NMR Center, National Institute of Chemistry, Hajdrihova 19, Ljubljana, Slovenia

[5]EN-FIST Center of Excellence, Trg OF 13, Ljubljana, Slovenia

[6]Faculty of Chemistry and Chemical Technology, Večna pot 113, Ljubljana, Slovenia

[7]Laboratory of Biophysics, Wageningen University & Research, Stippeneng 4, 6708WE Wageningen, The Netherlands

[8]MicroSpectroscopy Centre, Wageningen University & Research, Stippeneng 4, 6708WE Wageningen, The Netherlands

[9]Institute of Biochemistry, Biological Research Centre, Hungarian Academy of Sciences, Temesvári körút 62, H-6726 Szeged, Hungary

[*]Corresponding authors, G.G. (garab.gyozo@brc.mta.hu), H.V.As. (henk.vanas@wur.nl), P.H.L. (p.lambrev@gmail.com)

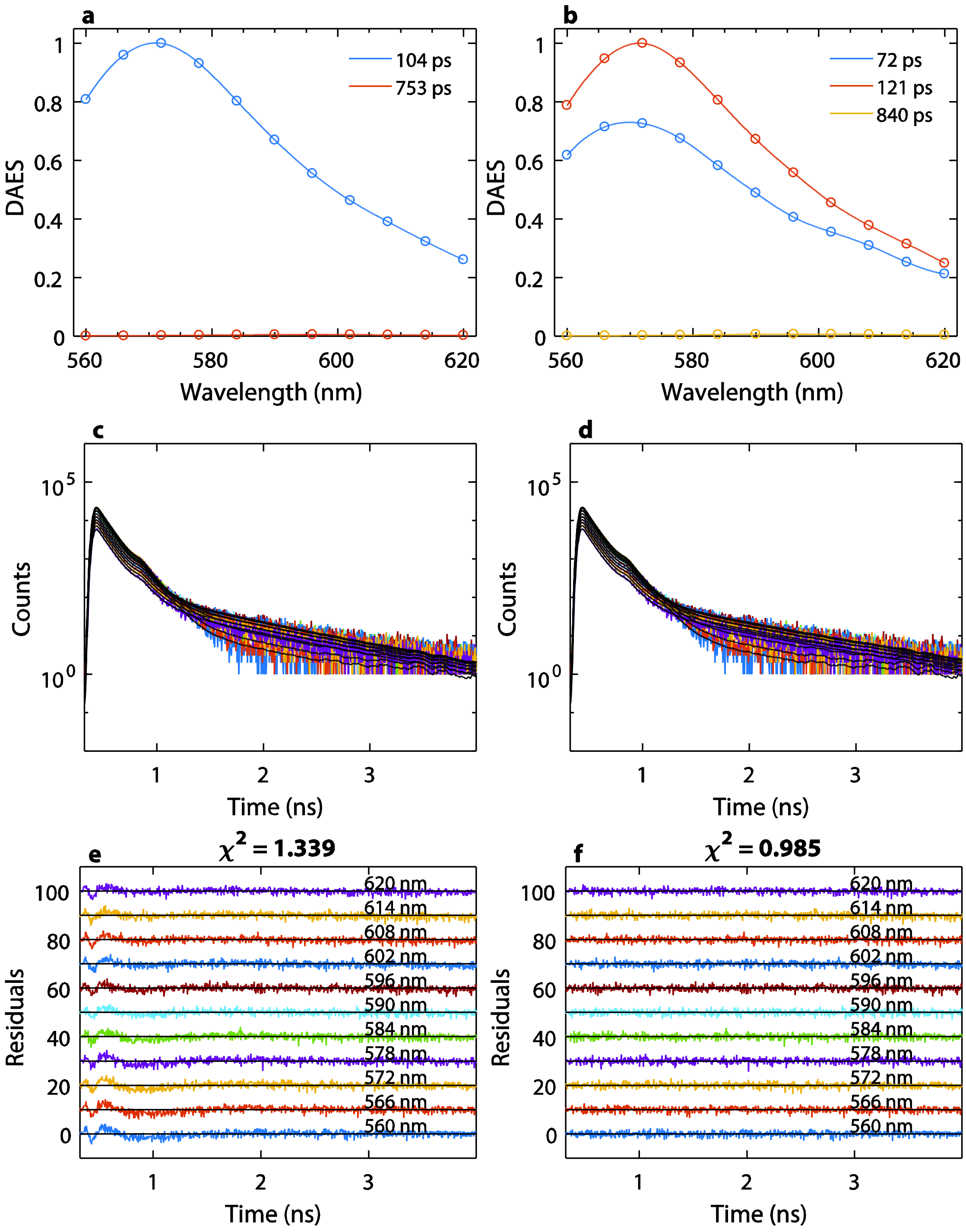

Supplementary Figure 1. Global lifetime analysis of the fluorescence decays of MC540 in resuspension buffer. Decay-associated emission spectra (DAES) (a, b), decay traces (c, d) and normalized residuals (e, f) obtained with a 2-component (a, c, e) and 3-component fit (b, d, f).

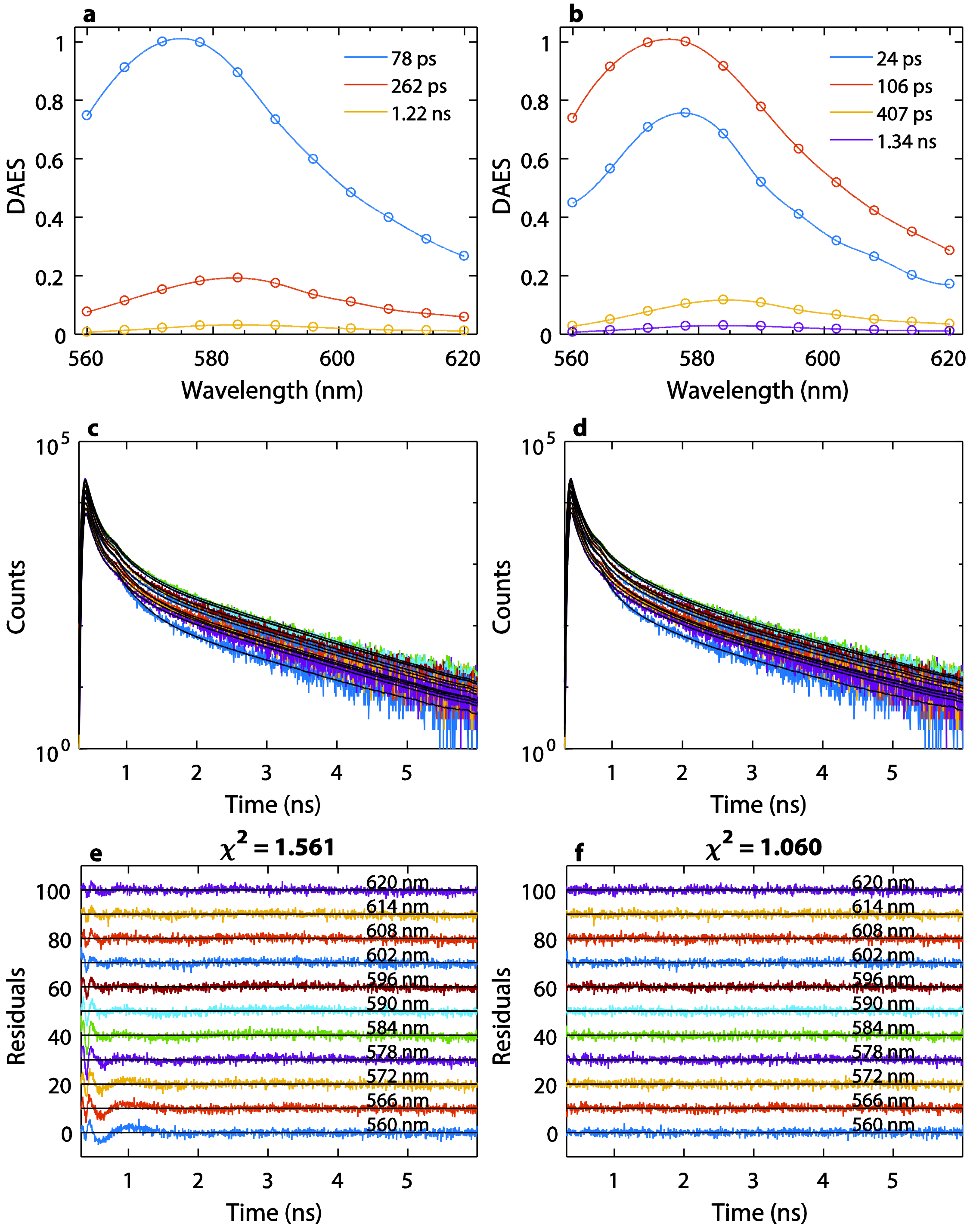

Supplementary Figure 2. Global lifetime analysis of the fluorescence decays of MC540 in thylakoid membranes. Decay-associated emission spectra (a, b), decay traces (c, d) and normalized residuals (e, f) obtained with 3-component (a, c, e) and 4-component fit (b, d, f).

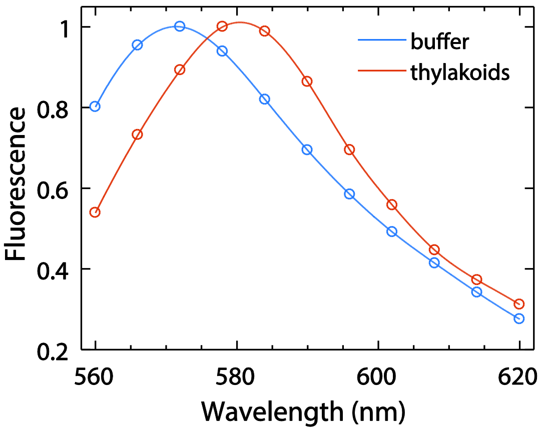

Supplementary Figure 3. Stationary fluorescence emission spectra of MC540 in resuspension buffer and thylakoid membranes.

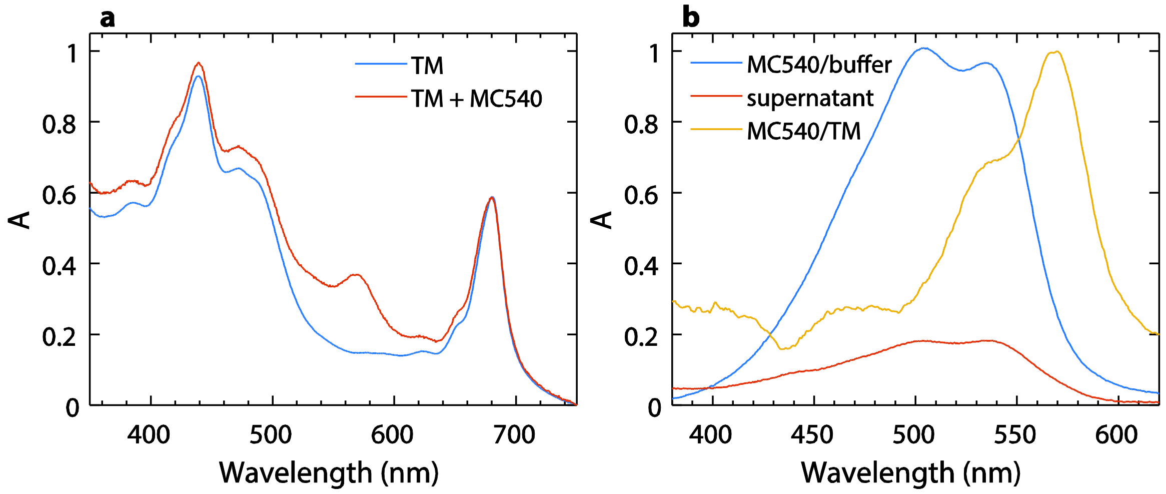

Supplementary Figure 4. Staining of thylakoid membranes with MC540. Absorption spectra of thylakoid membranes (TM) before and after incubation with 10 µM MC540 and subsequent washing (a); absorption spectra of 10 µM MC540 in resuspension buffer, of the supernatant after washing of thylakoid membranes, and difference spectrum of stained minus unstained thylakoid membrane (b). The spectrum of the supernatant shows that MC540 concentration is approximately 0.2 µM, which means that 80% of the dye is bound to the membranes. The difference spectrum shows the batochromic shift of the absorption peaks of MC540 in lipidic environment. The band around 500 nm, which is characteristic for MC540 dimers in water, is not observed when MC540 is bound to the thylakoid membranes.

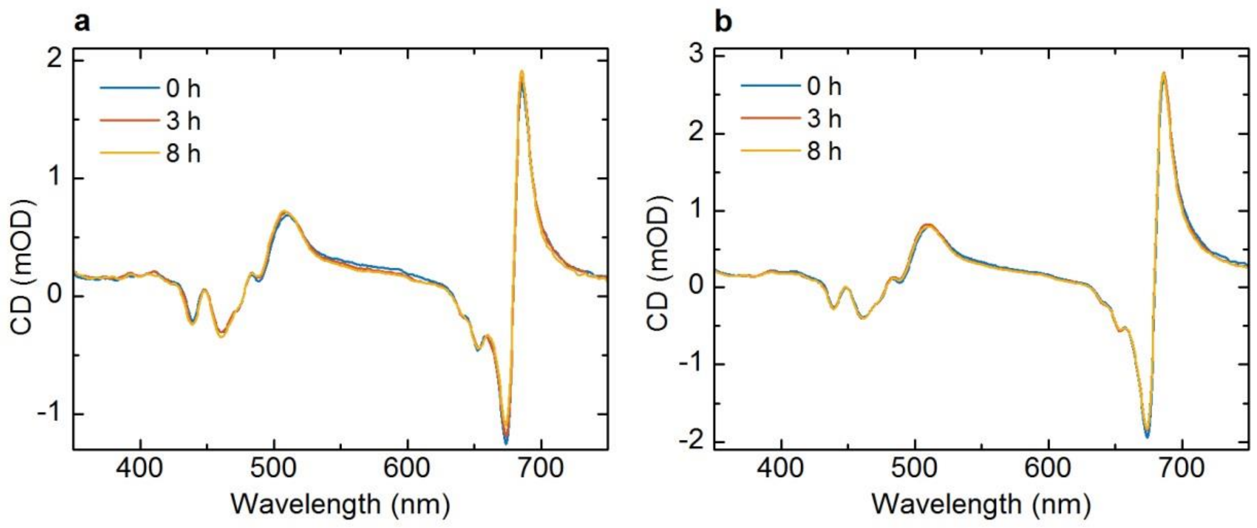

Supplmentary Figure 5. Circular dichroism spectra of spinach thylakoid membranes isolated and suspended in sorbitol-based (**a**) and NaCl-based (**b**) isotonic medium, as for the ^{31}P-NMR measurements. The spectra were collected immediately after the isolation of thylakoid membranes (0 h) and 3 and 8 hours later, as indicated, after storing the concentrated thylakoids (7.2 or 5.8 mg/ml chlorophyll a+b content, repectively) in the dark at 4 °C. The measurements were carried out at room temperature, as described earlier (Karlický et al. 2016), using a J 815 spectropolarimeter (JASCO, Tokyo, Japan). The spectra were recorded in steps of 0.5 nm with an integration time of 1 s, a band-pass of 2 nm and scanning speed of 100 nm min^{-1}. CD spectra of thylakoid membranes at concentration of 50 µg/ml chlorophyll (a+b) were recorded in a cell with an optical pathlength of 0.5 cm; the dilution was made immediately before the measurements.

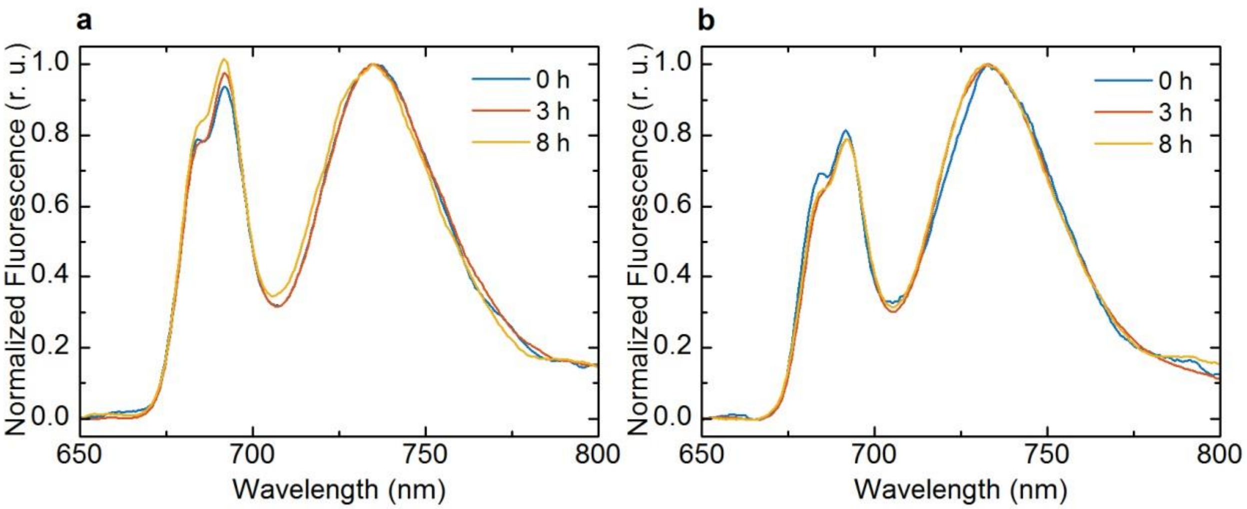

Supplementary Figure 6. Normalized 77 K fluorescence emission spectra of spinach thylakoid membranes isolated and suspended in sorbitol-based (a) NaCl-based (b) isotonic medium, as for the ^{31}P-NMR measurements. The spectra were collected immediately after the isolation of membranes (0 h) and 3 and 8 hours later, as indicated, after storing the highly concentrated thylakoids (7.2 or 5.8 mg/ml chlorophyll a+b content, repectively) in the dark at 4 °C. The measurements were carried out as described earlier (Karlický et al. 2016) using an LS50B luminescence spectrofluorometer (Perkin-Elmer, Beaconsfield, United Kingdom) equipped with a custom-made Dewar-type optical cryostat. The chlorophyll a+b content was set to 25 µg/ml to avoid reabsorption (OD_{680nm} < 0.1 with the optical pathlength of 0.1 cm); the dilution was made immediately before the measurements.

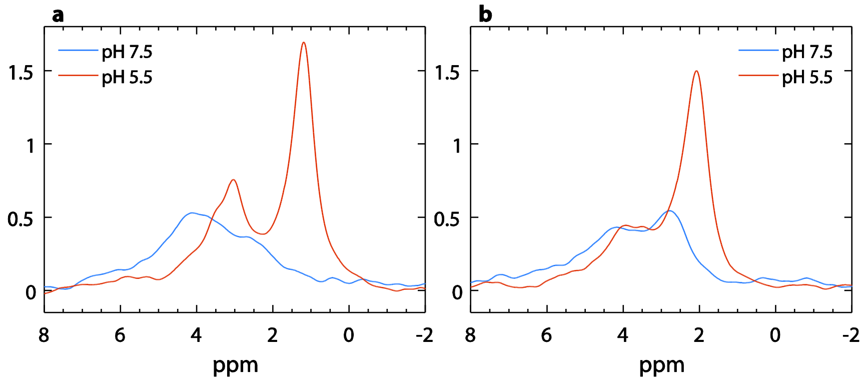

Supplementary Figure 7. Effect of low-pH on the ^{31}P-NMR peaks ascribed to the isotropic non-bilayer phases of spinach thylakoid membranes isolated and suspended in the sorbitol-based (a) and NaCl-based (b) medium. Chlorophyll a+b contents, pH 7.5: 9.8 mg/ml and pH 5.5: 13.1 mg/ml (a) and 11.0 mg/ml (pH 7.5) and 9.1 mg/ml (pH 5.5) (b). The spectra were recorded at 5 °C.

Supplementary Table 1. F_V/F_M, the ratio of variable to maximal chlorophyll-a fluorescence intensities, of spinach thylakoid membranes (TM) isolated and suspended in sorbitol-based (7.2 mg/ml chlorophyll a+b content) and NaCl-based (5.8 mg/ml chlorophyll a+b content) isotonic medium, as for the ^{31}P-NMR measurements. The fluorescence transients were measured immediately after the isolation of thylakoid membranes (0 h) and 1, 3, 6 and 8 hours later, as indicated, after storing the concentrated thylakoid suspension in the dark at 4 °C. Immediately before the measurements, aliquots were taken and diluted to 50 µg/ml chlorophyll a+b content. The measuerements were carried out at room temperature in a PAM 101/103 fluorometer equipped with the emitter-detector unit ED-101BL (H. Walz. Effeltrich. Germany): weak blue LEDs radiation was used for excitation of the F_0 minimum fluorescence level, then saturating light pulse of 0.8 s duration and incident photon flux density of approximately 5000 µmol photons m^{-2} s^{-1} was applied to reach F_M level; $F_V = F_M - F_0$. n=3, ±SD; only one sample was measured for freshly isolated thylakoids in sorbitol based medium; detection, >660 nm.

Time after the isolation of TM	F_V/F_M	
(h)	Sorbitol-based medium	NaCl-based medium
0	0.815	0.658 ± 0.010
1	0.820 ± 0.001	0.657 ± 0.005
3	0.816 ± 0.001	0.668 ± 0.002
6	0.810 ± 0.002	0.683 ± 0.001
8	0.811 ± 0.002	0.674 ± 0.009

Supplementary Table 2. Fatty acid distribution of isolated spinach thylakoid membranes before (Control) and after catalytic homogen hydrogenation of the membranes (Hydrogenated).

**Fatty acids**	**Control**	**Hydrogenated**
16:0	8.3	9.5
16:1	3.2	5.3
16:3	8.3	5.8
18:0	1.3	5.7
18:1	2.0	17.7
18:2	6.4	14.0
18:3	70.5	42.0
Hydrogenation %:		23.6

Reference

Karlický, V. et al. Enhanced thermal stability of the thylakoid membranes from spruce. A comparison with selected angiosperms. Photosynth. Res. 130, 357-371 (2016).
